# Changes in plant nutrient status following combined elevated [CO_2_] and canopy warming in winter wheat

**DOI:** 10.3389/fpls.2023.1132414

**Published:** 2023-02-22

**Authors:** Jianqing Wang, Lianqing Li, Shu Kee Lam, Xiuzhen Shi, Genxing Pan

**Affiliations:** ^1^ Key Laboratory for Humid Subtropical Eco-geographical Processes of the Ministry of Education, Institute of Geography, Fujian Normal University, Fuzhou, China; ^2^ Institute of Resource, Ecosystem and Environment of Agriculture, College of Resources and Environmental Sciences, Nanjing Agricultural University, Weigang, Nanjing, China; ^3^ Faculty of Veterinary and Agricultural Sciences, The University of Melbourne, Parkville, Melbourne, VIC, Australia

**Keywords:** climate change, free-air CO2 enrichment (FACE), global warming, nutrient dynamic, winter wheat, Southeast China

## Abstract

Projected global climate change is a potential threat to nutrient utilization in agroecosystems. However, the combined effects of elevated [CO_2_] and canopy warming on plant nutrient concentrations and translocations are not well understood. Here we conducted an open-air field experiment to investigate the impact of factorial elevated [CO_2_] (up to 500 μmol mol^-1^) and canopy air warming (+2°C) on nutrient (N, P, and K) status during the wheat growing season in a winter wheat field. Compared to ambient conditions, soil nutrient status was generally unchanged under elevated [CO_2_] and canopy warming. In contrast, elevated [CO_2_] decreased K concentrations by 11.0% and 11.5% in plant shoot and root, respectively, but had no impact on N or P concentration. Canopy warming increased shoot N, P and K concentrations by 8.9%, 7.5% and 15.0%, but decreased root N, P, and K concentrations by 12.3%, 9.0% and 31.6%, respectively. Accordingly, canopy warming rather than elevated [CO_2_] increased respectively N, P and K transfer coefficients (defined as the ratio of nutrient concentrations in the shoot to root) by 22.2%, 27.9% and 84.3%, which illustrated that canopy warming played a more important role in nutrient translocation from belowground to aboveground than elevated [CO_2_]. These results suggested that the response of nutrient dynamics was more sensitive in plants than in soil under climate change.

## 1 Introduction

Increasing atmospheric CO_2_ concentration ([CO_2_]) is a major driver of climate change. Carbon dioxide is projected to reach more than 500 ppm, which may raise global temperature by more than 2°C by the end of this century (IPCC, 2021). It has been widely reported that elevated [CO_2_] stimulates terrestrial plant growth through the [CO_2_] fertilization effect (Wang et al., 2019; Chen et al., 2022), and indirectly affects nutrient absorption and availability (Lam et al., 2012a; Wang et al., 2018a). It is generally accepted that crop yield was increased through enhancing leaf photosynthesis under elevated [CO_2_] (Zhang et al., 2013; Wang et al., 2020b), while warming reduced crop biomass due to increased phenological development and leaf respiration (Ruiz-Vera et al., 2013; Venugopalan et al., 2021). By contrast, the high isoflavone reductase-like gene expression mitigated the negative impacts of elevated temperature in wheat (Shokat et al., 2021). However, crop responses to factorial elevated [CO_2_] and warming have not been sufficiently addressed.

Chinese agroecosystems are vulnerable to global climate change. Wheat is one of the most widely planted staple agricultural food crops (Nagai and Makino, 2009), but its yield could be severely affected by elevated [CO_2_] and global warming (Ray et al., 2015; Wang et al., 2019). Soil nutrient dynamics and plant nutrient absorption are critical factors determining the impact of elevated [CO_2_] and warming on food productivity (Wheeler and von Braun, 2013; Wang et al., 2020a; Wei et al., 2021). Previous reviews concluded that elevated [CO_2_] increased the average grain yield of C3 grasses (wheat, rice, and barley) by about 19% (Kimball, 2016). However, the negative impact of warming can negate the positive effect of elevated [CO_2_] on crop productivity (Ruiz-Vera et al., 2013; Wang et al., 2019). Whereas, Shokat et al. (2021) reported that high isoflavone reductase-like gene expression promoted grain yield under the combined treatment of elevated [CO_2_] and heat stress in wheat. Furthermore, the negative impact of warming played an overwhelming role in plant nutrient utilization in comparison with elevated [CO_2_] (Wang et al., 2019). To date, it remains unclear whether the impact of elevated [CO_2_] offsets the effect of canopy warming on nutrient transfer and soil nutrient status.

Nitrogen (N), phosphorus (P), and potassium (K) are recognized as the most limiting factors affecting the crop physiological function and production. Elevated [CO_2_] reduced nutrient concentrations owing to the dilution effect of [CO_2_] or inhibited investment in Rubisco and nitrate (Bloom et al., 2010; Ainsworth and Long, 2021), but increased nutrient accumulation in plants (Wang et al., 2018a). Furthermore, a large-scale meta-analytic study showed that the nutrient (N, P, K) declined in foliar and grain tissues under elevated [CO_2_] (Loladze, 2014). Meanwhile, warming accelerated leaf transpiration and more nutrients were acquired for aboveground biomass (Wang et al., 2018b). As a consequence, more nutrients are translocated from soil to plants (Viciedo et al., 2021). Nutrient availability was not only determined by soil properties, but also regulated by elevated [CO_2_] and global warming (Wang et al., 2016; Osanai et al., 2017). It is likely that changes in plant physiological metabolism can alter the translocation and accumulation of nutrients, which could finally affect soil nutrient dynamics under future climate changes (Calleja-Cabrera et al., 2020). Loladze (2002) reported that elevated [CO_2_] led to a global imbalance of essential elements in plants, and could intensify malnutrition in human populations under future climate conditions. Ma et al. (2007) showed that elevated [CO_2_] decreased the availability of N and P in a FACE (free-air [CO_2_] enrichment) system, but the response of nutrient availability to elevated [CO_2_] varied among nutrients and growth stages. Some studies reported that the availability of P and ammonium ( ${\text{NH}}_{4}^{+}$ -N) increased under elevated [CO_2_] or combined with warming (Bhattacharyya et al., 2014), although elevated [CO_2_] and warming decreased soil nutrient status in terrestrial ecosystems (Jauregui et al., 2015). So far, the factorial combination of elevated [CO_2_] and canopy warming on belowground processes and ecosystem functioning remains elusive.

This study was a factorial elevated [CO_2_] (to 500 ppm) and warming (by 2°C) experiment conducted in an open-field system. We hypothesize that both elevated [CO_2_] and warming would increase nutrient requirement, and thus stimulate nutrient translocation from belowground to aboveground, finally decreasing soil nutrient availability. The findings of this study provide insights into fertilizer management in cropland and strategies for sustainable production under future climates.

## 2 Materials and methods

### 2.1 Experimental design

The experimental site was located in Jiangsu Province, China (31°30′N, 120°33′E). The soil is formed on clayey lacustrine deposits as Gleyic Stagnic Anthrosol. Initial soil analysis was 19.2 g organic C kg^-1^, 1.3 g total N kg^-1^, 0.9 g total P kg^-1^, 15.0 g total K kg^-1^and pH of 7.0. The soil at the experimental site is classified as loam with sand of 33.8%, silt of 38.6%, and clay of 27.6%. The experimental site belongs to a humid subtropical climate with an average annual temperature of 16 °C and annual precipitation of 1100-1200 mm (Wang et al., 2018b).

The facility operation followed the procedures described by Wang et al. (2019). The treatments were randomly arranged in three blocks, with each block having four treatments (rings). The area of each ring is 50 m^2^. The four treatments included ambient condition (CK), elevated [CO_2_] (500 ppm, CE), canopy warming (+2°C, WA) by infrared heaters, and combined treatment of elevated [CO_2_] and canopy warming (CW). An interval of 28 m was set up between rings to avoid any potential contamination across treatments.

### 2.2 Crop cultivation and fertilizer management

Winter wheat (*Triticum aestivum* L.) of Yangmai No.14 was planted at a row spacing of 20 cm in November 2013 and harvested in May 2014. Based on local practice, the basal fertilizer in the form of urea (46% N) was applied at a rate of 187.5 kg ha^-1^, and top-dressed at a rate of 150 kg ha^-1^ at the elongation stage. The topdressing fertilizer was provided by a compound fertilizer (15 N: 15 P_2_O_5_: 15 K_2_O) at a rate of 375 kg ha^-1^ after the heading stage. The weed control and insecticide application were carried out according to local agronomic management. The wheat was cultivated under rain-fed conditions.

### 2.3 Soil and plant collection and measurement

Plant and soil samples were taken at the elongation, heading, and ripening. Since climatic change treatment altered wheat development, soil and plant sampling was conducted based on the phenological stage. Wheat plants were randomly collected 1 m^2^ from each plot, plant sample was separated into shoot and root. Root samples were rinsed with water to get rid of the soil. Meanwhile, the shoot and root samples were rinsed and de-enzyme at 105°C for 0.5 h, and then oven-dried at 70°C for 48 h. All plant samples were ground to 0.25 mm. For soil samples, five soil cores (0-15 cm) were taken and then homogenized to form a mixed soil sample. After removing visible residues and stones, soil samples were passed through a 2 mm sieve and maintained at 4°C before analysis.

Soil available P was extracted with NaHCO_3_ and analyzed by a spectrophotometer (TU-1810, China). Soil available K concentrations were estimated using a flame photometer (FP6410, China) after extraction with 1 M ammonium acetate (NH_4_OAc). Soil ${\text{NH}}_{4}^{+}$ -N and ${\text{NO}}_{3}^{-}$ N concentrations were determined using a subsection flow analysis instrument (Skalar, Netherlands).

Plant shoot and root samples were pretreated with H_2_SO_4_-H_2_O_2_, and analyzed for N concentrations by the Kjeldahl digestion method. Phosphorus concentrations were determined by a spectrophotometer (TU-1810, China), and K concentrations by a flame photometer (FP6410, China).

### 2.4 Statistical analysis

Nutrient transfer coefficients were used to evaluate the impact of elevated [CO2] and canopy warming on the capacity of plant nutrient uptake (Wang et al., 2016). Nutrient transfer coefficients were estimated as:


$$\text{Nutrient transfer coefficients}=\frac{Shoot\;nutrient\;\left(NPK\right)\;concentrations}{Root\;nutrient\;\left(NPK\right)\;concentrations}$$

general linear mixed model (GLM) was used to detect the [CO_2_], warming and growth stage (main factor), with blocks treated as a random variable. Accordingly, a three-way analysis of variance was used to test the differences between treatments and the growth stage. The probability level (*P*< 0.05) was considered to be statistically significant. Statistical analyses were conducted by SPSS v.22.0 (IBM Corp., Armonk, NY, USA).

## 3 Results

### 3.1 Nutrient concentrations

**Figure 1:**
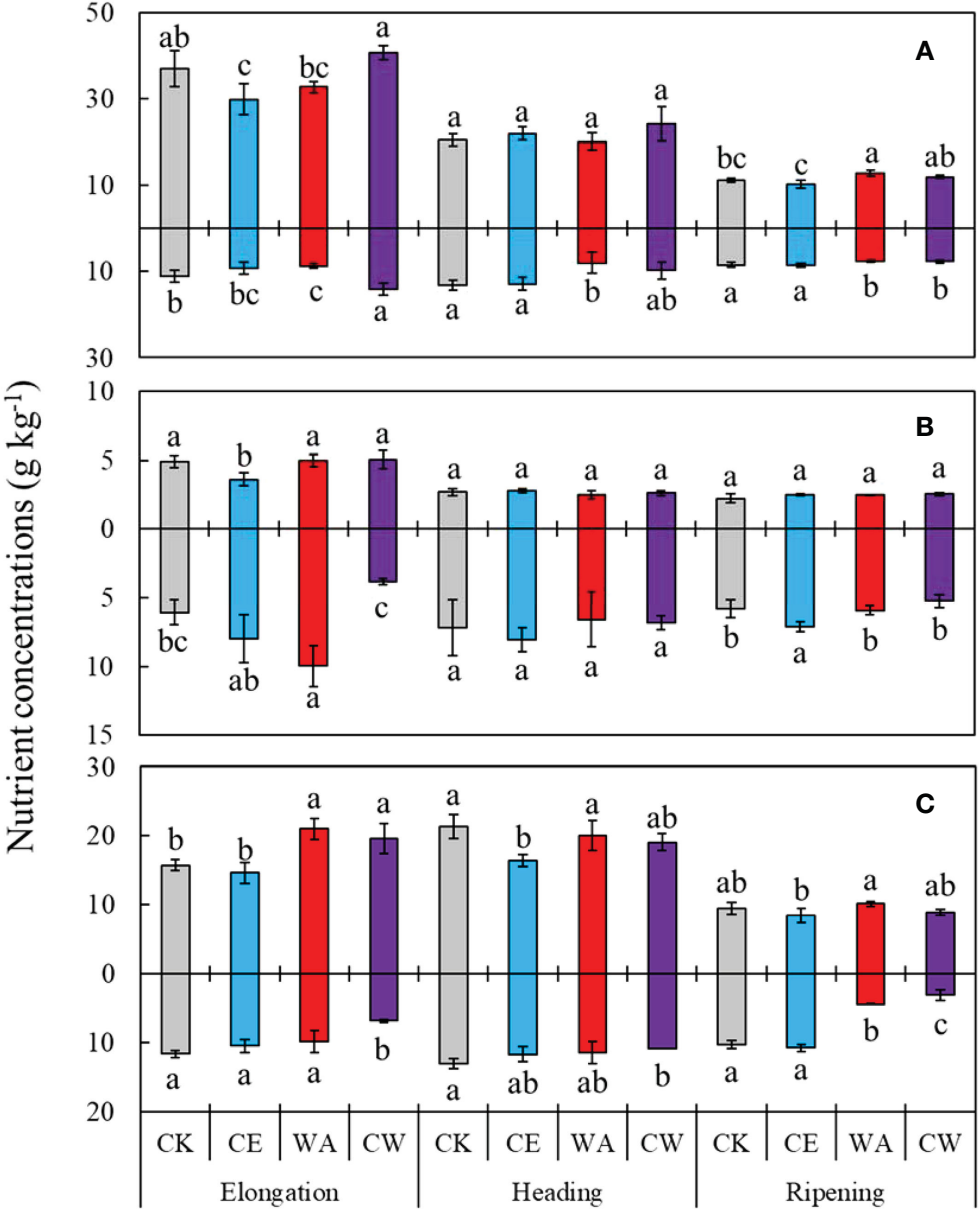
Nitrogen **(A)**, phosphorus **(B)**, and potassium **(C)** Concentrations in plant shoots (upper) and roots (lower) under ambient condition (CK), elevated [CO_2_] alone (CE), canopy warming alone (WA) and combined treatment (CW). Different letters indicate significant differences between treatments in the same stage at *P*< 0.05.

When averaged across three growth stages, elevated [CO_2_] did not alter plant N or P concentrations (**Figure 1**), but reduced K concentrations by 11.0% (*P* = 0.001) in the shoot and 11.5% (*P* = 0.001) in the root (**Figure 1** and **Table 1**). By contrast, canopy warming significantly increased shoot N, P, and K concentrations by 8.9% (*P* = 0.016), 7.5% (*P* = 0.054), and 15.0% (*P*< 0.001), but reduced root N and K concentrations by 12.3% (*P*< 0.01), and 31.6% (*P*< 0.001), respectively. However, plant N, P, and K concentrations varied among stages and reached the peak at the elongation stage. Significant interactions of [CO_2_] × warming and [CO_2_] × warming × stage were observed in the N and P concentrations: elevated [CO_2_] aggravated the positive effect of canopy warming on shoot N and P concentrations, especially at the elongation stage, but mitigated the adverse impact of canopy warming on root N and P concentrations at the heading stage.

**Table 1:** Summary of the GLMM analysis of nutrient concentrations in shoots and roots under simulated climate change conditions.

Source	df	N		P		K	
		Shoot	Root	Shoot	Root	Shoot	Root
[CO_2_] effect ^a^		3.2	8.8	-3.4	-5.9	-11.0	-11.5
Warming effect ^b^		8.9	-12.3	7.5	-9.0	15.0	-31.6
[CO_2_]	1	0.344	0.067	0.344	0.303	0.001	0.001
Warming	1	0.017	0.007	0.054	0.115	<0.001	<0.001
Growth stage	2	<0.001	<0.001	<0.001	0.051	<0.001	<0.001
[CO_2_]×Warming	1	0.001	0.002	0.114	<0.001	0.195	0.127
[CO_2_]×Stage	2	0.147	0.312	0.017	0.016	0.200	0.074
Warming×Stage	2	0.447	<0.001	0.009	0.638	<0.001	<0.001
[CO_2_]×Warming×Stage	2	0.001	0.006	0.019	0.001	0.102	0.148

a, The impact of elevated [CO_2_] was measured through ((CE +CW)/(CK+WA)-1) × 100, averaged across three stages;

b, The impact of canopy warming was measured through ((WA + CW)/(CK + CE)-1) × 100, averaged across three stages.

Different letters indicate significant differences between treatments in the same stage at P< 0.05.

### 3.2 Nutrient transfer coefficients

Elevated [CO_2_] and canopy warming had significant effects on nutrient transfer coefficients, and these effects varied with wheat growth stages (**Table 2**). When averaged across three growth stages, canopy warming significantly increased N, P, and K transfer coefficients by 22.2% (*P* = 0.001), 27.9% (*P* = 0.001), and 84.3% (*P*< 0.001), respectively. Elevated [CO_2_] enhanced P and K transfer coefficients by 27.9% (*P* = 0.058) and by 10.1% (*P*< 0.05), but did not affect the N transfer coefficient. A significant interaction of [CO_2_] × warming was detected for P (*P*< 0.001) and K (*P<* 0.01) transfer coefficients: the increases in P and K transfer coefficients by canopy warming were aggravated by elevated [CO_2_]. The impact of canopy warming appeared differently among stages as evidenced by significant warming × stage interaction.

**Table 2:** Nutrient transfer coefficients under ambient condition (CK), elevated [CO_2_] alone (CE), canopy warming alone (WA), and combined treatment (CW).

Growth stage	Treatment	N transfer coefficient (%)	P transfer coefficient (%)	K transfer coefficient (%)
Elongation	CK	334.6 ± 38.6ab	155.4 ± 2.9b	134.1 ± 6.9b
	CE	327.2 ± 44.2ab	172.6 ± 16.8b	120.8 ± 18.3b
	WA	380.3 ± 33.7a	270.0 ± 88.7a	169.1 ± 9.2a
	CW	291.9 ± 36.5b	251.3 ± 38.0ab	157.6 ± 12.0a
Heading	CK	81.8 ± 13.0b	39.4 ± 11.0a	38.2 ± 1.4b
	CE	45.9 ± 5.6c	35.2 ± 5.8a	35.2 ± 2.1b
	WA	51.0 ± 11.4c	42.1 ± 20.3a	41.7 ± 2.0b
	CW	130.5 ± 15.3a	38.4 ± 4.3a	48.8 ± 6.1a
Ripening	CK	135.5 ± 5.8c	164.8 ± 22.5a	92.0 ± 10.4c
	CE	140.2 ± 5.3c	141.3 ± 18.2a	78.4 ± 13.3c
	WA	218.1 ± 32.8b	176.1 ± 17.5a	231.4 ± 15.9b
	CW	287.6 ± 27.4a	176.2 ± 12.7a	297.3 ± 58.5a
[CO_2_] effect ^a^		-8.5	13.5	10.1
Warming effect ^b^		22.2	27.9	84.3
[CO_2_]		0.109	0.058	0.046
Warming		0.001	0.001	<0.001
Growth stage		<0.001	<0.001	0.016
[CO_2_]×Warming		0.130	<0.001	0.002
[CO_2_]×Stage		0.278	0.011	0.054
Warming×Stage		0.017	0.019	<0.001
[CO_2_]×Warming×Stage		0.397	<0.001	0.362

a, The impact of elevated [CO_2_] was measured through ((CE +CW)/(CK+WA)-1) × 100, averaged across three stages;

b, The impact of canopy warming was measured through ((WA + CW)/(CK + CE)-1) × 100, averaged across three stages.

Data were presented as means of three replicates ± standard error; Different letters indicate significant differences between treatments in the same stage at P< 0.05.

### 3.3 Soil nutrient status

Elevated [CO_2_] and canopy warming did not alter soil nutrient status (**Table 3**), although elevated [CO_2_] slightly increased soil available P concentrations by 13.2% (*P*< 0.05). However, soil nutrient availability varied across stages with the peaking soil ${\text{NO}}_{3}^{-}$ N concentrations occurring at the heading stage and the peaking P and K concentrations at the ripening stage. There was a significant interaction of [CO_2_] × warming × growing stages on soil K concentrations. A significant effect of elevated [CO_2_] × warming interaction was observed for soil ${\text{NO}}_{3}^{-}$ N and available P concentrations: warming increased soil ${\text{NO}}_{3}^{-}$ N and available P concentrations under ambient [CO_2_] but not under elevated [CO_2_].

**Table 3:** Soil nutrient status under ambient condition (CK), elevated [CO_2_] alone (CE), canopy warming alone (WA), and combined treatment (CW).

Growth stage	Treatment	${\text{NO}}_{3}^{-}$ N mg kg^-1^	${\text{NH}}_{4}^{+}$ -N mg kg^-1^	Available P mg kg^-1^	Available K mg kg^-1^
Elongation	CK	29.4 ± 0.2a	3.6 ± 1.1a	37.1 ± 1.1a	114.8 ± 13.7a
	CE	29.2 ± 0.7a	2.3 ± 0.3b	43.9 ± 5.7a	101.7 ± 12.7a
	WA	28.7 ± 0.5a	2.3 ± 0.3b	38.2 ± 5.4a	119.0 ± 4.4a
	CW	29.0 ± 0.6a	2.8 ± 0.2ab	40.8 ± 3.7a	124.2 ± 3.6a
Heading	CK	33.9 ± 15.5a	2.9 ± 0.1a	30.1 ± 3.9a	97.3 ± 3.1a
	CE	28.2 ± 14.6a	2.4 ± 0.2bc	37.1 ± 5.0a	98.3 ± 5.5a
	WA	47.2 ± 7.5a	2.4 ± 0.3c	39.4 ± 1.9a	100.7 ± 9.7a
	CW	31.5 ± 3.7a	2.9 ± 0.3ab	32.0 ± 5.4a	110.5 ± 12.6a
Ripening	CK	14.7 ± 7.4a	2.3 ± 0.4a	38.2 ± 5.6a	112.2 ± 7.7b
	CE	16.4 ± 5.0a	3.0 ± 0.6a	55.1 ± 3.4a	153.2 ± 4.2a
	WA	18.0 ± 0.9a	2.3 ± 0.1a	41.1 ± 9.3a	121.2 ± 19.5b
	CW	22.5 ± 5.1a	2.7 ± 0.1a	44.9 ± 8.4a	121.0 ± 17.3b
[CO_2_] effect ^a^		-8.8	2.1	13.2	6.6
Warming effect ^b^		16.6	-7.3	-2.2	2.8
[CO_2_]		0.307	0.697	0.011	0.058
Warming		0.095	0.169	0.630	0.392
Growth stage		<0.001	0.546	<0.001	<0.001
[CO_2_]×Warming		0.653	0.007	0.007	0.530
[CO_2_]×Stage		0.069	0.053	0.078	0.037
Warming×Stage		0.338	0.614	0.435	0.025
[CO_2_]×Warming×Stage		0.516	0.010	0.467	0.006

a, The impact of elevated [CO_2_] was measured through ((CE +CW)/(CK+WA)-1) × 100, averaged across three stages;

b, The impact of canopy warming was measured through ((WA + CW)/(CK + CE)-1) × 100, averaged across three stages.

Data were presented as means of three replicates ± standard error; Different letters indicate significant differences between treatments in the same stage at P< 0.05.

## 4 Discussion

### 4.1 Canopy warming has stronger effects on nutrient concentrations and translocations than elevated [CO_2_] in winter wheat

Elevated [CO_2_] alone decreased plant K concentrations, but the responses varied among growth stages (**Table 1**). The dilution effect in the K concentrations has been widely described in plants under elevated [CO_2_] (Han et al., 2015; Wang et al., 2019). Kanowski (2001) found that elevated [CO_2_] (790 ppm) reduced K concentrations in Flindersia. By contrast, elevated [CO_2_] did not alter the N and P concentrations for both shoot and root (**Figure 1** and **Table 1**). This contrasts with other studies and indicates that elevated [CO_2_] is associated with the dilution of nutrient concentrations in wheat grain under sufficient fertilizer input (Lam et al., 2012b). Our previous study argued that elevated [CO_2_] did not affect plant nutrient concentrations under adequate fertilizer supply in the rice paddy field (Wang et al., 2018a). On the other hand, the reasons were ascribed to the levels of [CO_2_] elevation (500 ppm) in this study, which was much lower than in other studies (more than 550 ppm). Whereas, the P and K transfer coefficients were significantly increased by elevated [CO_2_], which was ascribed to an increase in nutrient demand by crop aboveground biomass (Wang et al., 2019). Indeed, we observed that elevated [CO_2_] significantly increased grain yield by 29.6% (**Figure S1**). Elevated [CO_2_] did not affect the N transfer coefficient, which is due to the inhibition of ${\text{NO}}_{3}^{-}$ N assimilation or lower investment in Rubisco in the shoots of wheat (Bloom et al., 2010; Ainsworth and Long, 2021). These results indicated that the mechanisms for nutrient translocation from root to shoot varied with plant nutrient demands.

Canopy warming significantly increased nutrient concentrations in plant shoots (**Figure 1** and **Table 1**). Warming-induced increase in plant N concentrations (29.8-32.7%) was also observed in a tallgrass prairie ecosystem (An et al., 2005). Trueman and Gonzalez-Meler (2005) have indicated that higher air temperature would increase the vapor pressure deficit of the canopy and leaf transpiration, thereby increasing nutrient translocation from root to shoot. Our previous study showed a significant increase in evapotranspiration under canopy warming conditions in this winter wheat field (Wang et al., 2018b). Moreover, we also found that canopy warming significantly reduced nutrient concentrations in roots and increased nutrient transfer coefficients (**Tables 1**, **2**).

Our results observed that combined treatment of elevated [CO_2_] and canopy warming did not affect shoot nutrient concentrations (**Figure 1**). This is consistent with a previous study conducted by Cheng et al. (2010), who observed no significant change in rice N concentrations under concurrent elevated [CO_2_] (680 ppm) and high night temperature (+10°C). However, Jauregui et al. (2015) has shown that the interaction of elevated [CO_2_] (700 ppm) and temperature (+ 4°C) significantly decreased N and K concentrations, but did not affect the P concentrations in wheat leaf in a greenhouse study. In contrast, elevated [CO_2_] (550 ppm) and elevated air temperature (+ 2°C) significantly increased P uptake in rice in an open top chamber study (Bhattacharyya et al., 2014). The inconsistent results were attributed to differences in experimental designs and variations in crop cultivars, and the low statistical power of individual studies. Reich et al. (2016) found that temperature determined the response of plant N assimilation and mineral nutrient composition to elevated [CO_2_]. Similarly, our results found that canopy warming altered nutrient uptake response to elevated [CO_2_], with elevated [CO_2_] significantly increasing P and K transfer coefficients under canopy warming, but decreasing the P transfer coefficient under ambient temperature. Therefore, further studies are needed to reveal the mechanisms of plant nutrient assimilation under future concurrent elevated [CO_2_] and warming conditions.

### 4.2 Effects of elevated [CO_2_] and canopy warming on soil nutrient status

As mentioned above, elevated [CO_2_] and canopy warming significantly altered nutrient uptake. However, opposite to our hypothesis, elevated [CO_2_] or canopy warming did not affect soil nutrient status (**Table 3**). Previous studies reported that elevated [CO_2_] did not change soil N or P availability in paddy fields (Ma et al., 2007; Cheng et al., 2016), although it is generally accepted that elevated [CO_2_] increased nutrient demand through increasing plant biomass (Lam et al., 2012b; Wang et al., 2018a). However, a recent study demonstrated that [CO_2_] fertilization increased N and P availability in a P-limited forest ecosystem (Hasegawa et al., 2016). The present study was not constrained by nutrients due to the frequent fertilizer applications, which suggests that soil nutrient availability can be replenished by fertilizer input in an intensively managed agricultural ecosystem under future climate scenarios.

Our results showed that canopy warming increased soil ${\text{NO}}_{3}^{-}$ N and available P concentrations under ambient conditions (**Table 3**). Warming greatly affects soil microbial and enzyme activity, which stimulates soil nutrient availability (Liu et al., 2015; Osanai et al., 2015; Wang et al., 2016). Warming increased nutrient mineralization, which leads to the stimulation of nutrient availability and increases nutrient assimilation by plants (Zuccarini et al., 2020; Iversen et al., 2022). In contrast, canopy warming did not significantly alter soil nutrient availability under elevated [CO_2_] (**Table 3**). The acceleration of soil nutrient availability is counteracted by the plant demand and soil moisture, and was even reduced under warming. Elevated temperature decreased soil moisture, resulting in a limitation in soil nutrient mineralization under dry conditions (Borken and Matzner, 2009; Wang et al., 2016). Our results demonstrated that both canopy warming alone and combined with elevated [CO_2_] generally increased nutrient transfer coefficients (**Figure 1** and **Table 2**). Therefore, the long-term climatic change probably increases soil nutrient consumption, which has a negative impact on food production. However, the responses of soil nutrient status to future concurrent elevated [CO_2_] and canopy warming are complicated, which warrants further studies for developing adaptation strategies to future climate change.

## 5 Conclusion

Our results demonstrated that simulated climate change has a significant influence on nutrient concentrations and transfer coefficients. Canopy warming rather than elevated [CO_2_] increased N, P, and K concentrations in plant shoots, but reduced these concentrations in plant roots. Canopy warming significantly increased nutrient transfer coefficients. A similar trend was observed for nutrient transfer coefficients under elevated [CO_2_], but to less extent than canopy warming. This study demonstrated an increase in nutrient consumption under climate change in winter wheat. Our findings provide major implications for plant and soil nutrient management as affected by future climate change.

## Data availability statement

The original contributions presented in the study are included in the article/**Supplementary material**. Further inquiries can be directed to the corresponding authors.

## Author contributions

JW performed the laboratory work, analyzed the data and drafted the manuscript. XS revised and improved the draft. SL improved the draft. GP and LL contributed ideas to the study and carried out the experimental design. All authors contributed to the article and approved the submitted version.
