# Supplementary material for: Changes in plant nutrient status following combined elevated [CO2] and canopy warming in winter wheat
Source: Front Plant Sci. 2023 Feb 22;14:1132414. doi: 10.3389/fpls.2023.1132414 (PMC9992424; doi:10.3389/fpls.2023.1132414)
Supplement: Supplementary file 1 [file DataSheet_1.docx]

**Supplementary Material**

**Table S1** Nutrient (NPK) concentrations in shoots and roots under ambient condition (CK), elevated [CO_2_] alone (CE), canopy warming alone (WA) and combined treatment (CW).

| Growth stage | Treatments | N (g kg^-1^) | |  | P (g kg^-1^) | |  | K (g kg^-1^) | |
| --- | --- | --- | --- | --- | --- | --- | --- | --- | --- |
|  |  | Shoot | Root |  | Shoot | Root |  | Shoot | Root |
| Jointing | CK | 37.07±2.43ab | 11.13±0.79b |  | 4.90±0.27a | 6.07±0.54bc |  | 15.74±0.47b | 11.61±0.28a |
|  | CE | 29.83±2.08c | 9.22±0.86bc |  | 3.60±0.26b | 7.98±1.00ab |  | 14.58±0.88b | 10.39±0.55a |
|  | WA | 32.75±0.69bc | 8.64±0.30c |  | 4.99±0.24a | 9.98±0.85a |  | 21.02±0.88a | 9.78±0.92a |
|  | CW | 40.65±0.95a | 14.04±0.78a |  | 5.02±0.38a | 3.84±0.12c |  | 19.61±1.24a | 6.81±0.09b |
| Heading | CK | 20.55±0.80a | 13.24±0.65a |  | 2.69±0.15a | 7.19±1.17a |  | 21.35±0.98a | 13.02±0.43a |
|  | CE | 22.01±0.91a | 12.84±0.90a |  | 2.81±0.09a | 8.08±0.51a |  | 16.35±0.50b | 11.66±0.62ab |
|  | WA | 20.16±1.11a | 7.98±1.41b |  | 2.51±0.17a | 6.60±1.15a |  | 20.01±1.25a | 11.43±0.93ab |
|  | CW | 24.25±2.33a | 9.76±1.18ab |  | 2.62±0.12a | 6.84±0.29a |  | 19.06±0.74ab | 10.82±0.03b |
| Ripening | CK | 11.3±0.25bc | 8.44±0.31a |  | 2.22±0.19a | 5.79±0.39b |  | 9.38±0.50ab | 10.22±0.36a |
|  | CE | 10.29±0.59c | 8.57±0.28a |  | 2.50±0.04a | 7.12±0.23a |  | 8.36±0.57b | 10.72±0.31a |
|  | WA | 12.86±0.42a | 7.60±0.12b |  | 2.46±0.01a | 5.92±0.17b |  | 10.07±0.24a | 4.36±0.07b |
|  | CW | 12.01±0.2ab | 7.64±0.20b |  | 2.55±0.06a | 5.25±0.27b |  | 8.85±0.25ab | 3.07±0.42c |

Different letters indicate significant differences between treatments in the same stage at *P* < 0.05.

**
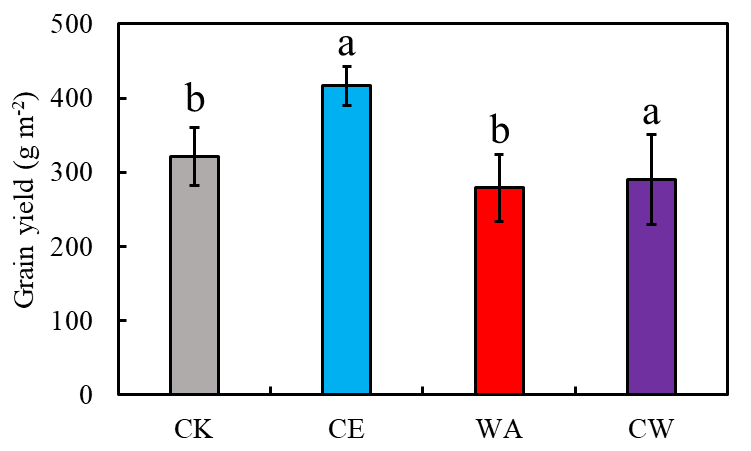
Fig. S1** Grain yield under ambient condition (CK), elevated [CO_2_] alone (CE), canopy warming alone (WA) and combined treatment (CW).

The data was cited from previous study by Wang et al. (2016).

**References**

Wang, J., Liu, X., Zhang, X., Smith, P., Li, L., Filley, T.R., Cheng, K., Shen, M., He, Y. & Pan, G. (2016) Size and variability of crop productivity both impacted by CO_2_ enrichment and warming—A case study of 4 year field experiment in a Chinese paddy. Agriculture, Ecosystems & Environment, 221, 40-49.
